# A cold-blooded culprit: Salmonella enterica subsp. arizonae pulmonary infection in an immunocompromised patient: a case report

**DOI:** 10.1099/acmi.0.001107.v3

**Published:** 2026-01-30

**Authors:** Sarah Kenny, Hassan Cheaito, Sadhbh Mc Loughlin, Niamh Reidy, Susan Lapthorne, Tim Healy, Aileenn O' Connor, Aaron Doherty, Jennifer Walsh, Catiriona Hickey, John Luke Kiely, Gerard Daniel Corcoran

**Affiliations:** 1 Department of Microbiology, Cork University Hospital, Cork, Ireland; 2 Department of Respiratory, Mallow General Hospital, Cork, Ireland

**Keywords:** Immunocompromised host Non-typhoidal Salmonella

## Abstract

**Background.** *Salmonella enterica* subsp. *arizonae* is an uncommon zoonotic pathogen primarily associated with reptiles. While most infections are gastrointestinal, invasive infections such as bacteraemia, osteomyelitis, meningitis and septic arthritis have been reported, particularly in immunocompromised individuals. Pulmonary infections are exceedingly rare.

**Case presentation.** A 66-year-old woman with rheumatoid arthritis on a tumour necrosis factor-alpha inhibitor presented with a 2 week history of progressive cough and dyspnoea. She reported prolonged exposure to a pet snake. Sputum cultures confirmed *S. enterica* subsp. *arizonae*, with susceptibility to ciprofloxacin. Imaging revealed the right lower lobe infiltrate without cavitation. She was successfully treated with ciprofloxacin for 2 weeks, with resolution of symptoms.

**Conclusion.** This case highlights an uncommon presentation of *Salmonella* infection in an immunocompromised patient with bronchiectasis. With increasing exotic pet ownership, clinicians should maintain a high index of suspicion for *Salmonella enterica* subsp. *arizonae* in patients with known reptile exposure.

## Data Summary

All data associated with this work is reported within the article.

## Introduction

*Salmonella enterica* subsp. *arizonae* is an uncommon cause of human infections and is primarily associated with reptile exposure, particularly snakes, turtles and lizards. While non-typhoidal *Salmonella* serotypes are frequently linked to gastroenteritis, *Salmonella arizonae* has been increasingly recognized as a cause of invasive infections, particularly in immunocompromised hosts.

Reptiles serve as a primary reservoir for *S. arizonae*, and human transmission typically occurs through direct contact with reptiles, contaminated enclosures, or ingestion of contaminated food or water. The first reports of reptile-associated *Salmonella* infections date back to the 1930s, when infections in lizards and other reptiles were documented as potential zoonotic threats. Notably, *S. arizonae* has a unique predilection for reptilian hosts, making it a distinct cause of reptile-associated salmonellosis in humans.

Immunocompromised individuals, especially those receiving tumour necrosis factor-alpha (TNF-α) inhibitors for autoimmune diseases such as rheumatoid arthritis, inflammatory bowel disease and psoriasis, are at increased risk of severe opportunistic infections. TNF-α plays a critical role in macrophage activation and granuloma formation, and its suppression impairs host defence mechanisms against intracellular pathogens, including *Salmonella*, *Mycobacterium tuberculosis* and *Listeria monocytogenes*.

This report presents a rare case of pulmonary *S. arizonae* infection in a patient receiving TNF-α inhibitor therapy and with prolonged reptile exposure, emphasising the need for heightened clinical awareness of zoonotic risks in immunocompromised populations.

## Case presentation

### Clinical history and presentation

A 66-year-old woman with a history of bronchiectasis and rheumatoid arthritis, managed with monthly golimumab injections for 5 years, presented with a 2-week history of worsening cough, increased sputum production and exertional dyspnoea. She denied fever, night sweats, weight loss or gastrointestinal symptoms.

She reported close exposure to a pet corn snake over a prolonged period, which she had owned for 15 years. She frequently handled the reptile and cleaned its enclosure without gloves. There was no recent travel history, exposure to sick individuals or contact with farm animals.

### Physical examination and initial findings

On examination, her vital signs were unremarkable: temperature 36.8 °C, heart rate 82 beats per minute, blood pressure 120/78 mmHg, respiratory rate 16 breaths per minute, and oxygen saturation 97% on room air. Respiratory examination revealed mild coarse crackles over the right lower lung field. No lymphadenopathy, hepatosplenomegaly or cutaneous lesions were noted.

### Microbiological and radiological investigations

Initial laboratory tests showed raised inflammatory markers, with a white cell count of 18.3×10⁹ l^−1^ and C-reactive protein of 64 mg l^−1^, consistent with active infection.

Sputum culture on MacConkey agar demonstrated non-lactose-fermenting colonies, and xylose lysine deoxycholate agar showed hydrogen sulphide negative colonies, prompting consideration of atypical *Salmonella*. Identification using MALDI-TOF mass spectrometry (Bruker) yielded a confidence score of 2.28, confirming *S. enterica*. Serotyping using poly-O and poly-H antisera (O4, O5 and O9 negative; Hi, Hgm and Henx negative) produced an antigenic formula of 41:z4,z23:-, consistent with *S. enterica* subsp. *arizonae*. Whole-genome sequencing (BioNumerics v8.1) identified the strain as sequence type 2131.

A culture of bronchoalveolar lavage (BAL) also isolated *S. arizonae*, confirming pulmonary involvement. Blood cultures were sterile. Review of previous respiratory cultures showed no prior isolation of *Salmonella* species or other Gram-negative pathogens, supporting this as a new infection rather than colonization. Antimicrobial susceptibility testing using Etest (bioMérieux) was performed and read according to EUCAST v15.0 methodology [1] with *Escherichia coli* ATCC 25922 as the quality-control strain. MICs (mg l^−1^) were as follows:

**Ciprofloxacin:** 0.047 mg l^−1^ (susceptible)

**Ceftriaxone:** 0.064 mg l^−1^ (susceptible)

**Meropenem:** 0.032 mg l^−1^ (susceptible)

**Azithromycin:** 12 mg l^−1^ (interpretation per EUCAST NTS guidance)*

**Trimethoprim–sulfamethoxazole:** 0.125 mg l^−1^ (susceptible)

*EUCAST does not provide routine azithromycin breakpoints for *S. enterica*; interpretation contextualized based on NTS guidelines.

A chest X-ray revealed mild right lower lobe infiltrate. Computed tomography of the thorax confirmed a small right lower lobe consolidation without cavitation.

### Clinical course

Targeted antibiotic therapy with oral ciprofloxacin 500 mg twice daily was started after identification of *S. arizonae*. Given the patient’s immunocompromised status and the unusual site of infection, a 2-week course was completed. Temporary discontinuation of golimumab was advised to reduce the immunosuppressive burden during the acute phase of infection. The patient responded well to treatment with resolution of the exacerbation. Following medical advice, she agreed to permanently remove the snake from her household to eliminate ongoing zoonotic risk and recurrent infection.

## Discussion

This case underscores the growing importance of recognizing zoonotic infections, particularly those caused by *S. enterica* subsp. *arizonae*, in immunocompromised individuals. *S. arizonae* is primarily associated with reptiles, particularly snakes, lizards and turtles. It was first isolated and described by Caldwell and Ryerson in 1939 from snakes in the Southwestern USA [2]. Historically, *S. arizonae* infections were most commonly reported in AZ, CA and other regions of the Southwestern USA, corresponding to areas with high reptile populations [3]. Although rare compared to other non-typhoidal *Salmonella* serovars, *S. arizonae* remains an important zoonotic pathogen, especially with the increasing popularity of exotic pet ownership. Human infections typically arise through direct reptile contact, contaminated enclosures or ingestion of contaminated food or water.

The patient in this case had prolonged exposure to a pet corn snake for over 15 years and regularly handled the reptile without gloves. While she had no prior history of zoonotic infections, her use of the TNF-α inhibitor golimumab placed her at higher risk for opportunistic infections. Patients using TNF-α inhibitors are at an increased risk of infections, including those caused by *S. arizonae*, due to the immunosuppressive effects of these agents. TNF-α is crucial for the formation and maintenance of granulomas, which are essential for containing intracellular pathogens like *Salmonella*. Inhibition of TNF-α disrupts granuloma integrity, leading to increased susceptibility to both new infections and reactivation of latent infections [4,5].

It is interesting to note that the patient had owned the same pet corn snake for over 15 years, yet only developed *S. arizonae* infection after 5 years of immunosuppressive therapy. This delayed onset may be explained by the intermittent nature of *Salmonella* shedding in reptiles, which can fluctuate depending on factors such as stress, illness or poor husbandry conditions. This highlights the unpredictable risk posed by long-term reptile ownership in immunocompromised individuals, even when no prior infections have occurred.

*S. enterica* subsp. *arizonae* is most frequently associated with gastrointestinal infections, typically presenting as mild to moderate gastroenteritis, particularly following reptile exposure [6]. A case series from Taiwan demonstrated that bacteraemia and gastrointestinal symptoms were the predominant clinical manifestations among adult patients infected with *S. arizonae* [7]. In immunocompromised individuals, however, *S. arizonae* demonstrates a propensity for invasive disease, including bacteraemia, osteomyelitis, septic arthritis, meningitis and urinary tract infections [8,9]. Osteomyelitis and septic arthritis often require prolonged antimicrobial therapy and sometimes surgical intervention, while urinary tract infections and meningitis caused by *S. arizonae* have been linked to significant morbidity, particularly in neonates and elderly patients. These diverse clinical manifestations highlight the broad pathogenic potential of *S. arizonae*, particularly among vulnerable patient populations.

Pulmonary infections caused by *S. arizonae* are exceedingly rare but have been documented, particularly in immunocompromised patients. Table 1 summarizes the previously published cases, along with the present case.

**Table 1.** Summary of previous published case of *S. arizonae* associated pulmonary infections

Study	Year	Country	Specimen source	Patient condition	Susceptibility	Treatment	Outcome
Casado *et al*. [15]	1997	Spain	Sputum	HIV infection	Sensitive to ceftriaxone and ciprofloxacin	Ceftriaxone → Ciprofloxacin (oral)	Recovered
Baranzelli *et al*. [16]	2017	France	BAL fluid	COPD, asthma, ABPA, diabetes	Sensitive to amoxicillin-clavulanate, cephalosporins	Amoxicillin- clavulanate	Recovered
Shima *et al*. [17]	2017	Japan	Pleural fluid	SLE and lymphoma	Sensitive to ceftriaxone, levofloxacin	Ceftriaxone → Levofloxacin (oral)	Recovered
Current case	2025	Ireland	Sputum +BAL	Rheumatoid arthritis on TNF-α inhibitor	As above	Ciprofloxacin	Recovered

ABPA, allergic bronchopulmonary aspergillosis; BAL, bronchoalveolar lavage; COPD, chronic obstructive pulmonary disease; HIV, human immunodeficiency virus; SLE, systemic lupus erythematosus; TNF-α, tumour necrosis factor-alpha.

The recovery of *S. arizonae* from sputum and BAL cultures in the setting of an acute exacerbation of her bronchiectasis supported a diagnosis of *Salmonella* infection rather than incidental colonization. Imaging studies revealed a localized right lower lobe infiltrate without cavitation, suggesting a focal bacterial pneumonia. Negative blood cultures and the absence of systemic illness or sepsis further supported the diagnosis of localized pulmonary infection.

While haematogenous spread is the recognized route for extraintestinal *Salmonella* infection in immunocompromised patients, this case suggests that an alternative mechanism such as aspiration or haematogenous seeding is at play here. Structural lung disease, such as bronchiectasis, may also have contributed to infection susceptibility.

Another important consideration in *S. arizonae* infections is rising antimicrobial resistance. Wódz *et al*. reported the isolation of multidrug-resistant *S. diarizonae* from a mallard duck [10]. Additionally, Lamas *et al*. found that *S. arizonae* exhibits biofilm-forming capabilities that contribute to environmental persistence and antimicrobial resistance [11]. Fortunately, in this case, antimicrobial susceptibility testing showed that the *S. arizonae* isolate was susceptible to ciprofloxacin, ceftriaxone, meropenem and trimethoprim/sulfamethoxazole, allowing successful treatment with a 2-week course of ciprofloxacin.

Patient counselling played a critical role in addressing ongoing risk and preventing reinfection. Given her long-standing attachment to the pet snake, the decision to rehome the reptile was a difficult but necessary step to reduce future zoonotic risks. The Centers for Disease Control and Prevention (CDC) and multiple public health organisations recommend that immunocompromised individuals, young children and the elderly avoid direct contact with reptiles due to the risk of *Salmonella* transmission [12]. Several case reports highlight instances where inadequate hygiene measures following reptile handling have led to severe infections in at-risk individuals. Proper patient education on hand hygiene, enclosure cleaning and avoidance of direct reptile contact with the face or mouth is critical in mitigating these risks.

Environmental sources of *S. arizonae* should also be considered when evaluating zoonotic infections. Evangelopoulou *et al*. demonstrated that *S. arizonae* has been isolated from pork meat [13], suggesting that transmission may not be limited to direct reptile contact. Additionally, Crespo *et al*. found that *S. arizonae* was present in integrated turkey operations [14], raising concerns about its potential spread within the food industry. The ability of *S. arizonae* to persist in food sources and form biofilms suggests that human infections may occur through ingestion of contaminated food products, in addition to direct reptile exposure. The presence of *S. arizonae* in both reptilian and foodborne reservoirs highlights the need for ongoing surveillance to identify potential food-related outbreaks and reduce transmission risks.

The increasing number of case reports describing *S. arizonae* infections in non-endemic regions suggests that this pathogen may be more widespread than previously thought. With globalization and the exotic pet trade expanding, the potential for cross-border transmission of reptile-associated *Salmonella* infections is increasing. Public health efforts should focus on educating pet owners, veterinarians and healthcare providers about the risks associated with reptile ownership, particularly in households with immunocompromised individuals. Future research should explore the genetic mechanisms underlying *S. arizonae* virulence and resistance, as well as potential interventions to reduce transmission risks in both pet and food industry settings.

## Conclusion

This case demonstrates a rare occurrence of pulmonary *S. arizonae* infection in an immunosuppressed patient with prolonged reptile exposure. Given the rising prevalence of exotic pet ownership, clinicians must remain vigilant for zoonotic infections in at-risk populations. Early diagnosis, targeted antimicrobial therapy and patient education on preventive measures are essential for successful management and long-term infection control. Further research is needed to understand the full clinical spectrum of *S. arizonae* infections, transmission dynamics and strategies to reduce zoonotic transmission.

## Abbreviations

BAL: bronchoalveolar lavage
TNF-α: tumour necrosis factor-alpha
